# Nutrition Care for Patients with Weight Regain after Bariatric Surgery

**DOI:** 10.1155/2013/256145

**Published:** 2013-11-19

**Authors:** Carlene Johnson Stoklossa, Suneet Atwal

**Affiliations:** Nutriton Service, Alberta Health Services, Seventh Street Plaza, 10030-107 Street, Edmonton, AB, Canada T5J 3E4

## Abstract

Achieving optimal weight outcomes for patients with obesity is important to the management of their chronic disease. All interventions present risks for weight regain. Bariatric surgery is the most efficacious treatment, producing greater weight losses that are sustained over more time compared to lifestyle interventions. However, approximately 20–30% of patients do not achieve successful weight outcomes, and patients may experience a regain of 20–25% of their lost weight. This paper reviews several factors that influence weight regain after bariatric surgery, including type of surgery, food tolerance, energy requirements, drivers to eat, errors in estimating intake, adherence, food and beverage choices, and patient knowledge. A comprehensive multidisciplinary approach can provide the best care for patients with weight regain. Nutrition care by a registered dietitian is recommended for all bariatric surgery patients. Nutrition diagnoses and interventions are discussed. Regular monitoring of weight status and early intervention may help prevent significant weight regain.

## 1. Introduction

Obesity is a chronic disease that presents significant challenges for treatment long term. For lifestyle interventions, only 20% of people attempting weight loss are able to achieve and maintain 5% weight loss over a year [1]. Factors that predict weight regain after weight loss include a loss of >15–30% of initial weight, early weight regain, and not responding to early regain [2]. Interventions and strategies for weight regain that have been found effective in the nonsurgical literature include self monitoring, continued patient-provider contact, and increased physical activity [3].

In order to determine weight regain, it is important to understand the definition of weight stability. A stable weight is not just one number on the scale; it is normal to see some small fluctuations in weight. Weight stability has been defined as ±5 kg for both surgical and nonsurgical patients [4, 5]. Weight regain could then be identified when the weight has increased beyond the weight stable range.

Weight outcomes are better with surgical interventions. “Success” in terms of weight outcomes in the surgical literature has been described as 50–75% excess weight loss (% EWL), 20–30% loss of initial weight, and achieving a BMI less than 35 kg/m^2^ [6–12]. Weight regain is a risk for all patients after bariatric surgery. Approximately 20–30% of patients with bariatric surgery do not achieve successful weight outcomes [7, 8]. Regain of 20–25% of the lost weight after bariatric surgery can occur over a period of 10 years [8]. “Failure” in terms of weight outcomes can then be described as <50% EWL, <20% loss of initial weight, and a BMI ≥ 35 kg/m^2^. It is challenging to accurately determine the incidences of weight regain in the postbariatric surgery population due to the loss of patients to follow up over the long term and differences in reporting weight trends. 

There are several factors that influence weight outcomes, including weight regain after bariatric surgery. The purpose of this paper is to review some of the key factors and their nutrition implications. 

### 1.1. Type of Surgery

Weight outcomes differ by surgical procedure. In one meta-analysis they found that the sleeve gastrectomy (SG), compared to the laparoscopic adjustable gastric band (LAGB), achieved higher % EWL and greater improvement with diabetes [13].

Compared to malabsorptive procedures, restrictive procedures tend to have higher rates of weight loss failure and weight regain [12, 14–16]. In the study by Romy et al. [9], surgical failure was defined as EWL < 25% or BMI > 35 kg/m^2^ or a need for reversal/conversion procedure. The outcomes from this study indicated that LAGB had a higher failure rate compared to roux-en-Y gastric bypass (RYGB) (see Table 1). Both maximum weight lost and rate of weight loss were better with RYGB.

### 1.2. Food Tolerance

There is a lower food tolerance in bariatric surgery patients compared to nonsurgical obese patients [17]. Food tolerance results from a quality of eating questionnaire indicated that all bariatric surgery patients, regardless of procedure, reported problems with food tolerance in the short term (3–6 months), indicating the need for nutrition monitoring and evaluation. For RYGB patients, food tolerance is reduced after 3 months but then becomes comparable to non-surgical obese population at one year. However, for LAGB patients at 6 months they had a lower food tolerance compared to all other procedures, and this tolerance worsened over 5 years [17, 18]. In another study it was found that on top of more food intolerances, LAGB patients also have more frequent vomiting [9]. Since LAGB patients have more food tolerance issues that persist overtime (>12 months), they may require more monitoring [18]. 

It is possible to regain weight even with food intolerances, as energy intake can exceed needs. Foods that improve satiety (protein and fibre) and have lower caloric density (raw fruits and vegetables) may not be well tolerated after bariatric surgery. For example, a patient may not tolerate a whole raw apple (1 medium = 72 calories) but may tolerate apple juice (1 cup 250 mL = 118 calories) easier [19]. By switching from a solid texture to a liquid texture, a larger portion can be consumed which may increase caloric intake and contribute to weight gain over time.

Signs and symptoms that a patient is experiencing low food tolerance may include food selection that is limited to liquid or pureed texture, limited food variety, vomiting after intake of solid foods, swallowing difficulty (feeling of food “getting stuck”), consumption of liquids with solids, nausea, abdominal pain and cramping, and heartburn/reflux. 

Depending upon the amount of tolerance and food selection, nutritional status can be compromised. There is a risk of protein malnutrition due to low food tolerance related to the texture of some high protein foods, such as red meat or well cooked/dry meats or poultry. Low intake of fruits and vegetables can contribute to low fibre intake and vitamin/mineral intake. A registered dietitian can assess nutritional status and help with management of food tolerances. 

### 1.3. Energy Requirements

Nutrition prescription is an important component of nutrition intervention of weight management. It can be quite challenging to determine energy (calorie) requirements. Predictive equations are commonly used; however, the accuracy can vary and do not account for body composition which can impact energy requirements [20, 21]. There are reductions in energy requirements during and shortly after weight loss [4]. With the significant changes of both fat mass and fat free mass after bariatric surgery, it puts into question how much reduction in energy requirements occur with weight loss. 

Adaptive thermogenesis is a decrease in energy expenditure (EE) after weight loss beyond what can be predicted from changes in fat mass and fat free mass [22]. In a study with nonsurgical, obese subjects, those with larger weight loss showed a greater reduction in measured resting metabolic rate (RMR) compared to predicted values [23]. Several studies have assessed metabolic rate in bariatric surgery patients, and they have found a decrease in RMR after surgery [23]. In patients with vertical banded gastroplasty, a reduction in sleeping metabolic rate persisted after adjustment for body composition, at both 12 months and 98 months as long as weight loss was maintained [24]. In RYGB patients who experience weight regain, it has been seen that their RMR is lower than the weight stable group and that predictive formulas overestimate their RMR [25]. Reduction in energy requirement is sustained as long as weight loss is maintained in both surgical and nonsurgical patients; for those who regain weight to or above their starting weight, adaptive thermogenesis is no longer observed [4, 24].

An important component of total daily energy requirements is energy expenditure. Physical activity is important for both weight and other health related outcomes; however, most Canadians do not meet the recommended levels of activity, regardless of BMI. In one particular study, only 1/3 of the surgical group reported engaging in a level of physical activity consistent with recommendations for prevention of weight regain, compared with 60% of the non-surgical group [5]. Regular activity after surgery is related to better % EWL, maintenance of loss and fat-free mass [25, 26]. The increase in total amount of activity from presurgery to postsurgery has been found to be more important than the intensity [26]. Overall, more physical activity is associated with better postoperative weight loss maintenance [27, 28].

A sustained adaptive thermogenesis favors positive energy balance and may predispose to weight regain [4]. Measurement of resting energy requirements would improve accuracy of nutrition prescriptions, and optimization of energy expenditure may be helpful to achieve energy balance to help prevent further weight regain

### 1.4. Drivers to Eat

There are multiple drivers that effect hunger, appetite, satiety, and reward; these drivers influence our eating behaviours. Susceptibility to cues that trigger overeating can increase weight regain regardless of initial weight loss method (surgical versus nonsurgical) [5].

Chapman et al. [29] identified television watching, alcohol intake, and sleep deprivation as drivers to eat in healthy individuals. Each behaviour was linked to disinhibition resulting in less restrictive food consumption and weight gain. When all 3 behaviors were present, the effect was stronger. Consistent and long term exposure to these drivers develops a conditioned effect and decreases ones capacity or tendency to exhibit inhibitory control. Higher levels of disinhibition and increases in disinhibition over 1 year are significant predictors of weight regain in both surgical and nonsurgical participants. At both National Weight Control Registry entry and 1-year followup, surgical participants reported lower dietary restraint than non-surgical participants [5]. 

In one study RYGB patients (>18 months post op) with weight regain reported a return of problematic presurgical eating behaviours, such as increased hunger, difficulty coping with the return of food cravings, and grazing on energy dense foods [30]. Odom et al. [31] identified independent predictors of significant weight regain after gastric bypass; these predictors were increased food urges, decreased well being, and concerns of alcohol or drug use.

Changes in energy intake may be also affected by alterations in gut and adipocyte hormones, including ghrelin, PYY, GLP-1, and leptin [32–35].

### 1.5. Errors in Estimating Intake

Self monitoring of intake is a key health behaviour to help support patients with self management. Dietitians use this information collected by the patients as part of their assessment. Self reported intake, on average, will underestimate actual intake by 20 to 50%. Underreporting impacts estimates of calories, macronutrients and micronutrients. Some studies have shown a greater underreporting for calories than protein [36, 37]. Problems with accuracy of self reported intake have been documented across all BMI categories, genders, and age groups [38–40]. In a study with 1551 adults, underreporting occurred in 75–88% of the adults [41]. In the NHANES 11 study (*n* = 11663), 31% of adults underreported dietary intake [42]. 

In the group of non-surgical, obese patients, although estimations of portion sizes were accurate, they recalled eating 20% less than they actually ate, resulting in underreporting of 40% of their calories. Higher degree of underreporting occurred with patients who reported less hunger and disinhibition [43]. Females, people with a higher BMI (>30 kg/m^2^), those with a high need for social desirability, and people with dietary restraint, tend to have higher rates of underreporting [40, 42, 44–47]. 

Influences on under-reporting of intake include both intentional and unintentional factors. Intentional underreporting may include not reporting foods usually consumed but avoided at the time of recording, not reporting foods consumed that are not consistent with their diet plan, or not reporting foods that the patient considers “hostile” or “bad” (i.e., sweets). Unintentional factors may include a lack of recording foods due to infrequency of intake or having a genuine lack of recall (i.e., forgetting snacks, beverages) [36, 48].

It is important to discuss with patient the inherent errors with self reported intake. Patients express great frustration when they review their food records and the calories are within target but the expected weight change does not occur. Calculations of the reported intake need to be discussed in context of these errors. To improve accuracy, dietitians can work with patients to find self monitoring tools that best suits their needs, improve estimation of portions, teach patients how to accurately measure food portions, and help patients access accurate sources for nutrition information. 

### 1.6. Adherence

Adherence to recommendations is important for any intervention to be successful. The frequency of followup will depend on the type of procedure and comorbid conditions. Nutrition care by a registered dietitian is recommended for all bariatric surgery patients. 

Adherence with follow-up visits after bariatric surgery improves weight outcomes. In particular to the LAGB, frequent followup for nutrition and band adjustments are important; missing <25% of appointments was associated with greater loss of excess body weight [10, 49, 50]. Dixon et al. [51] found that a follow-up frequency less of than 13 times in 2 years especially males was associated with less weight loss, and Weichman et al. [52] found that less than 7 follow-up visits per year was associated with less loss of excess body weight (both studies looked at LAGB patients). Frequency of post-surgery visits is also inversely related to weight regain after gastric bypass [31].

Although followup is recommended, adherence to post-surgery visits is low. In one study, 782 RYGB patients were followed over 5 years. At 4 years, data was available for 60% of these patients of which 64% experienced weight regain. Of those patients that experienced weight regain, 60% never had nutritional follow up and 80% never underwent psychological followup [53]. 

Adherence with self monitoring behaviours decreases likelihood of weight regain [31]. In the study by Stewart et al. [30], RYGB patients with weight regain were able to identify a number of behaviour change strategies that worked initially after surgery, but overtime relaxed their use of these strategies, including self monitoring (i.e., journaling, measuring weight) and attending follow-up appointments. 

Bariatric surgery can present many challenges impacting a patient's nutrition-related behaviours and oral intake. It can be difficult to comprehend and adhere to all recommendations and optimize nutritional status. Over time, there is a trend for increase in energy intake: 1500, 1700, 1800, 1900, and 2000 kcal/day, at 6 months and 1, 2, 3, and 4–10 years after surgery [4]. Adherence to nutrition appointments and recommendations improves outcomes. Nutrition counseling was effective in reducing the total body weight and body fat of RYGB patients (2 years post-op) with weight regain as per a study done by Faria et al. [56]. Del Corral et al. [57] investigated weight regain in non-surgical obese patients and found that higher adherence to a low energy diet predicted lower weight regain over 2 years; the high adherence group regained 50% of their weight loss compared with 99% in the low adherence group.

### 1.7. Food and Beverage Choices

Although bariatric surgery provides restriction to help limit food portions, energy intake can be high if foods with high caloric density are consumed. Caloric dense foods such as ice cream, nuts and nut butters, oils, high-fat condiments (mayonnaise, gravy), chocolate, and alcohol can contribute to excess calories even when consumed in smaller portions.

Consumption of caloric beverages contributes to total energy intake. Some patients have low awareness of caloric content of beverages or may not record caloric beverages as part of daily intake. The calories consumed in beverages do not provide the same satiety/fullness cues as solid food; therefore, compensation at the next meal does not occur [58]. In one study, replacement of caloric beverages with water or calorie-free beverages resulted in a weight loss of 2–2.5% in obese subjects [59].

In general, patients are recommended to eat 3 small meals plus 1 or two snacks each day. Some patients eat frequently or present with grazing pattern, which can contribute to exceeding caloric requirements. A greater percentage of surgical participants (compared to nonsurgical) reported night eating at least one time per week at both National Weight Control Registry entry and 1 year followup, and this may contribute to weight regain [5].

### 1.8. Patient Knowledge

Knowledge base of patients around bariatric surgery is quite variable. Multiple sources of information (i.e., health care provider, friend/family, colleague, media, and internet) vary in accuracy and applicability to that individual. Patients may not be aware that weight regain can occur. Their expectations for weight outcomes may be high and not consistent with outcomes in the published literature. 

Use an open and inquisitive approach. It is important to not assume a patient is “noncompliant” with recommendations as they may have not received appropriate and current evidenced-based information. Patients presenting with weight regain likely had their surgery a few years ago, and it is important to assess and update them with current evidence-based information. 

## 2. Nutrition Care

Assessment by a registered dietitian is recommended for all bariatric surgery patients [10, 60]. A comprehensive nutrition assessment should include a detailed food and nutrition-related history, measurements of height and weight, a detailed weight history (see Box 1), review of biochemical data, and a review of medical tests and procedures. Assessment of energy intake and adequacy of both macronutrient and micronutrient intake will determine the nutrition status of the patient. Energy requirements can be estimated with a predictive equation (i.e., Mifflin St. Jeor); however, a measured RMR is recommended. 

Nutrition diagnoses that could be appropriate for patients presenting with weight regain after bariatric surgery include Excessive Energy Intake (NI-1.3), Limited Food Acceptance (NI-2.9), Unintended Weight Gain (NC-3.4), Food-and-Nutrition Related Knowledge Deficit (NB-1.1), Self Monitoring Deficit (NB-1.4), and Undesirable Food Choices (NB-1.7) [61].

The nutrition recommendations after bariatric surgery are discussed within the published literature [3, 10, 54]. A summary of key points is provided in Box 2. A nutrition prescription includes total energy recommended to achieve weight loss and prevention of further weight gain, as indicated. Nutrition education is important to address any knowledge deficits and to provide strategies to achieve goals of care. Self monitoring of intake, activity, and weight is recommended. Records can include not just the foods consumed but also time and location of eating, and any triggers for eating (i.e., emotions or feelings). Counseling and support can help build self monitoring skills and strategies to overcome barriers and improve accuracy.

### 2.1. Coordination of Care

A comprehensive team approach can provide the best care for patients with chronic diseases, including patient with obesity and postbariatric surgery. Interventions for weight regain should include a multidisciplinary approach, including nutrition, physical activity, behavioural modification with frequent followup. Coordinate care with a physician to assess for medical reasons for weight gain (i.e., thyroid, medication). Mental health assessment and support may be indicated for significant problematic eating behaviours. Coordinate care with a surgeon to assess for mechanical or surgery-related complications (i.e., band slippage, LAGB fill volume, and stomal dilation) or a need for surgical revision [3, 10].

## 3. Conclusion

Weight regain after bariatric surgery demonstrates the chronic and progressive nature of obesity. It is a complex disease requiring expertise from many health care providers. Monitoring of weight by both patient and health care providers can identify when weight regain occurs. Weight gain of more than 5 kg can be used as indicator for early assessment and intervention. Early intervention for weight regain may help prevent excessive regain and obesity-related health concerns. Registered dietitians have an important role to optimize care for patients with weight regain by improving self management skills, prevention/treatment of nutritional deficiencies, and optimizing nutritional status [10, 54]. 

## Figures and Tables

**Box 1:**
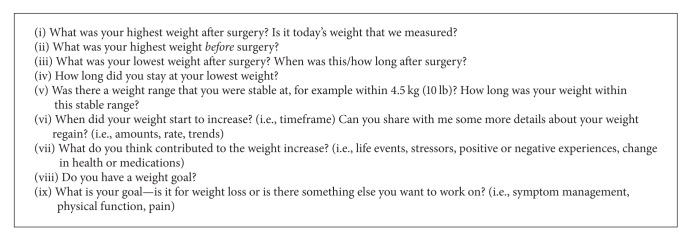
Suggested questions for a detailed weight history.

**Box 2:**
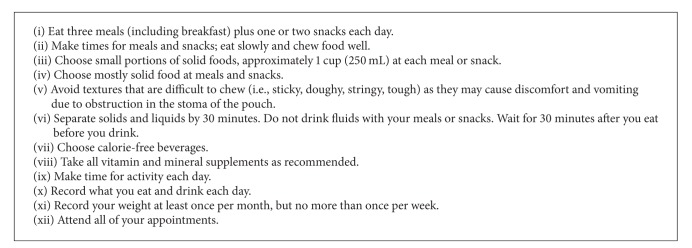
Summary of nutrition recommendations for patients after bariatric surgery [10, 54, 55].

**Table 1:** Surgical failure rate in LAGB versus RYGB [9].

	EWL < 25% or need for reversal/conversion procedure		BMI > 35 kg/m^2^ or need for reversal/conversion procedure	
	LAGB	RYGB	LAGB	RYGB
Three years	18.2%	0%	31.7%	6.9%
Six years	38.9%	2.5%	48.3%	12.3%
